# Experimental Research on Polymer-Based Coaxial Sealing Systems of Hydraulic Cylinders for Small Displacement Velocities

**DOI:** 10.3390/polym14020290

**Published:** 2022-01-11

**Authors:** Tudor Deaconescu, Andrea Deaconescu

**Affiliations:** Department of Industrial Engineering and Management, Transilvania University of Brasov, 500036 Brasov, Romania; deacon@unitbv.ro

**Keywords:** coaxial sealing systems, hydraulic cylinders, stick-slip, eigen-vibration, polyurethane, polytetrafluoroethylene

## Abstract

Reducing friction in the coaxial sealing systems of hydraulic cylinders is one of the solutions for increasing the energy efficiency of industrial actuations. This is a requirement, particularly in the case of small velocities that carry the risk of eigen-vibrations and/or stick-slip. The authors discuss the experimental research conducted on three coaxial sealing systems made from thermoplastic polymer and polyurethane type materials. The paper presents the equipment and method used for the experimental determination of static and kinematic friction coefficients and discusses the subsequent results obtained to test different working parameters. The experimentally determined friction coefficients yielded a range of materials recommended for coaxial seals such as to minimize the occurrence of jerky operation.

## 1. Introduction

Research on the energy efficiency of industrial actuation systems cited in the literature [1] shows a mere 21% efficiency of hydraulic drives, thus revealing an insufficiently optimized field. Despite their very low energy efficiency, hydraulic actuation systems have been deployed heavily in industrial applications that require large forces, rigidity and endurance. Reducing energy consumption in hydraulic drives can be achieved mainly by increasing the efficiency of the components. Thus, hydraulic cylinder efficiency can be improved by reducing the level of friction present in the system. Besides the large quantity of dissipated energy, friction also affects the control, precision and repeatability of the hydraulic cylinder’s motions.

Many applications of hydraulic cylinders entail small velocities of just several millimeters per minute. Included here are, for example, the feed motions of machine-tools, precise positioning motions by means of hydraulic cylinders or remote manipulation needed in applications on land, sea or in space.

It is known that for small velocities, in the case of dry friction, as well as of limit or mixed friction, the motion of the surfaces forming the so-called friction couple can be accompanied by intermittence or jolts. In the case of hydraulic cylinders, displacement of the mobile element at small velocities can cause two forms of jolty sliding, namely sliding accompanied by eigen-vibrations and intermittent sliding, known as stick-slip [2]. Sliding accompanied by eigen-vibrations is characterized by small amplitude variations of the friction force and passing from its maximum to its minimum values within a relatively large interval of time. Stick-slip is characterized by large amplitudes of the oscillatory phenomenon and large friction forces. Passing from maximum to minimum values takes place in very short time intervals, causing high frequency jerking. The sliding velocity at that stick-slip occurs is smaller than that typical for eigen-vibrations [3].

Non-uniform, jerky and uncontrolled motions at small velocity operation of hydraulic cylinders has numerous causes, including deviations from coaxiality of piston and rod, lack of component manufacturing precision, air pockets in the hydraulic oil and most importantly the inadequate selection of the sealing system. Hydraulic cylinder sealing system performance is influenced by numerous variables that cannot be simultaneously taken into consideration for obtaining generalized mathematical relationships. Speeds or properties of the sealing system components materials, roughness of the contacting surfaces, and temperature of the sealed fluid are only a few such quantities that affect the operational behavior of sealing systems [4,5,6]. The importance of an optimum sealing system design follows also from the conclusion of the study published by Oprean et al. [7], which asserts that 44% of the operational malfunctioning of hydraulic cylinders is due to flawed sealing.

While the literature features numerous reports on hydraulic cylinder behavior at small velocities, such research concerns the particular applications of sealing systems and the respective results do not lend themselves for generalization. Thus, a study by Eclipse Engineering Inc. presents the effects of stick-slip on seals (softening, swelling, and premature wear) as well as means of prevention (correct selection of materials, optimum roughness of the contacting surfaces, suitable fluids deployed at adequate temperatures) [8].

Sealing systems manufacturer, Trelleborg Sealing Solutions of Sweden, addresses stick-slip from a pragmatic perspective and proposes introducing dampers in order to absorb vibrations. Thus, by integrating an elastomer element into the sealing system the micro-movements created by the stick-slip phenomenon at the sealing contact area can be dampened to eliminate vibration and noise [9].

Several scientific papers describe experimental set-ups used for studying the stick-slip phenomenon. Pan et al. present an experimental apparatus developed to measure the friction forces for hydraulic cylinders under different operating conditions [10]. Puglisi et al. also discuss in [11] the experimental identification of friction effects defined by the parameters of the LuGre model.

Research reported in [12] demonstrates that stick-slip is caused by the transfer of carbon monoxide from carbon steel to the sealing surfaces. This leads to noise and accelerates the wear of sealing elements.

Further numerous research papers focus on the selection of sealing system materials. In [13], the authors recommend the selection of certain seal materials by area of application (food and beverage or oil and gas applications). In [14], Tran et al. assert that the type of friction occurring in the sealed area depends on pressure and seal material.

Most frequently, the materials used for hydraulic cylinder sealing systems are polymers, predominantly elastomers, plastomers or thermoplastic elastomers. These materials meet best the multiple requirements for a sealing system, namely, to achieve the best possible packing while ensuring maximum energy efficiency and motion precision.

One of the polymer materials most frequently used for seals is PTFE (virgin polytetrafluoroethylene) [12,15,16] or filled PTFE [16,17]. Research showed that polytetrafluoroethylene presents a small friction coefficient (0.05 … 0.1) and is also nearly completely chemically inert to the substances it comes into contact with. These characteristics render PTFE eligible for tribological applications designed to reduce energy consumption in friction-intensive machinery, as well as for reactive and corrosive applications [4]. Another polymer material is polyurethane, which, when combined with certain solid lubricants, ensures small friction coefficients.

A study of the construction of a large number of linear hydraulic cylinders has revealed the increasing use of coaxial sealing systems made from improved antifriction materials. The catalogues of sealing system manufacturers show the almost complete replacement of classic elastomers by materials based on plastomers, thermoplastic elastomers or duromers, possessing quite remarkable antifriction qualities.

Within this context the paper presents the results of experimental research conducted on coaxial sealing systems of hydraulic cylinders for small displacement velocities. Seals made from virgin PTFE, PTFE 25% Glass and H-PU 55D (polyurethane), respectively, were tested in view of establishing the optimum working ranges (for velocities, pressures) for each type of material.

The second section of the paper describes the structure of a coaxial sealing system and the main characteristics of the utilized materials. The third section presents a study of the friction in the analyzed tribosystem, aimed at the experimental determination of the static and kinematic coefficients. The paper is completed by the conclusions and recommendations resulting from the study.

## 2. Coaxial Sealing System

**Figure 1:**
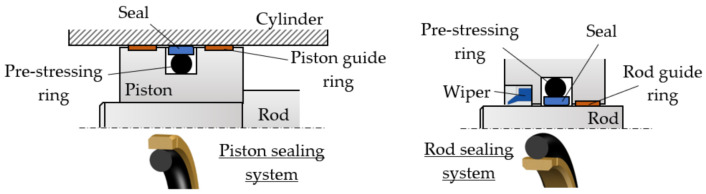
Coaxial sealing systems.

Coaxial sealing systems are widely used for both pistons and rods of hydraulic cylinders (Figure 1).

A coaxial sealing system consists of a seal that can have various cross-section forms and is in direct contact with the mobile surface and with a pre-stressing ring that can also have a number of different cross section geometries (circular, rectangular, complex). The pre-stressing rings do not engage in contact with the mobile surface and are, thus, not subject to wear; their role is merely to generate the pressure required for tensioning the entire system. Pre-stressing rings are also vibration dampers. A pre-stressing ring is mounted in its seat in pre-compressed state, with an initial specific radial deformation of 10–25%. As the pressure of the fluid in the hydraulic cylinder builds up, the deformation of the prestressing ring increases and generates a larger radial force.

The relative motion between the seal and the mobile surface of the hydraulic cylinder (the interior wall or the rod of the cylinder) causes the appearance in the contact area of a variable thickness fluid film that is wedge shaped. Depending on the velocity *v,* the thickness of the fluid film determines the type of friction between the seal and the surface of the cylinder, that can be dry, fluid or mixed. A greater velocity causes a thicker fluid film, and the consequent fluid type friction ensures a high level of energy efficiency. This is, however, conflicting in relation to the sealing process, as the hydrodynamic separation of seal and cylinder surface leads to an increased loss of fluid that flows towards the lower pressure side [4].

At small velocities the thickness of the fluid layer is also smaller, even zero, hence the type of friction will be dry. Responsible are the adhesion forces between the contacting materials and the asperities of the two surfaces. Dry friction must be avoided as it can cause uncontrolled motions of the hydraulic cylinder. This can be achieved by a correct selection of the seal materials.

Correct selection of the compounds for the sealing system components is significantly facilitated by knowing the chemical structure of the various materials and fluid that are used, as well as their reciprocal reactions.

The pre-stressing rings of coaxial sealing systems are generally made from elastomers of various types: nitrile (NBR), hydrogenated nitrile butadiene rubber (HNBR), silicone rubber (Q), fluorocarbon (FKM), ethylene propylene diene monomer (EPDM) and fluorosilicone (FVMQ). For high temperature working situations, up to 325 °C, the material recommended for these rings is perfluoroelastomer (FFKM), while for very low temperatures, as low as −75 °C, the recommended material is VMQ [4,18].

The second element of the sealing system, the actual seal, which is in contact with the mobile elements of the hydraulic cylinder, is made from plastomers of polytetrafluoroethylene (PTFE) type. This material is used as it combines a number of important properties, such as chemical inertness, ageing strength, the ability to fill the irregularities of the sealed-off surface and, mostly, the smallest friction coefficient in contact with various other solid materials.

Depending on the concrete operational conditions, seals are made from virgin PTFE or PTFE with added bronze, glass fibers, molybdenum disulfide (MoS_2_) or carbon fiber (graphite). Bronze endows the seal with increased extrusion strength, molybdenum disulfide facilitates low-friction and ensures long seal life, while the carbon fiber is responsible for a long wear life.

Another material used for seals is polyurethane, due to its resistance to high pressure and low friction coefficient. Variants of polyurethane compounds are filled with different solid lubricants and are used especially for dry running applications. Solid lubricants decrease the friction between the cylinder and the seal.

The performance of coaxial sealing systems at small linear velocities was tested for seals made from three different materials, Polytetrafluoroethylene (PTFE) and a self-lubricated thermoplastic polyurethane elastomer (H-PU). In all cases, the pre-stressing ring was an O-ring made from nitrile butadiene rubber (NBR) with a hardness of 70 Shore A. Table 1 shows the essential characteristics of the studied materials [19].

The analyzed coaxial sealing systems were mounted on the piston of a hydraulic cylinder with a barrel made from OLC45 steel (Romanian standard) (heat-treated quality carbon steel; DIN EN AISI 1.0503 C45-1045). For each of several tested polymer-steel pairs of materials the static and kinetic friction coefficients were determined. Consequently, recommendations are made for the range of velocities within that these seals can function without occurrence of the stick-slip phenomenon.

**Figure 2:**
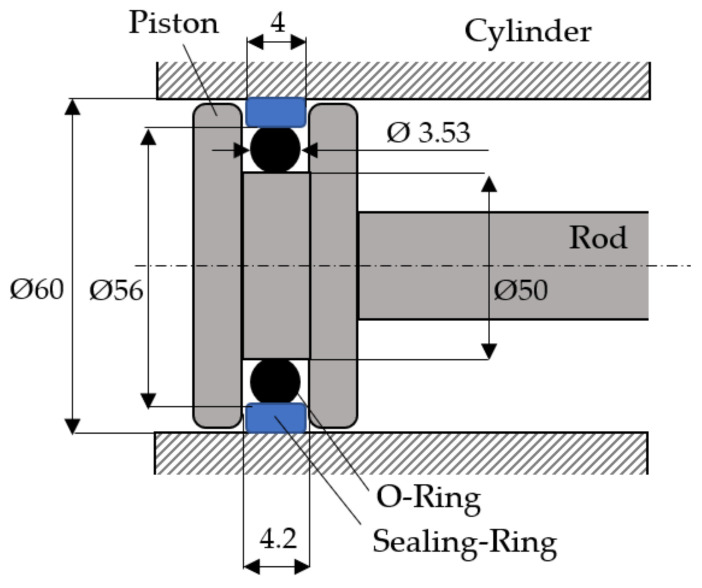
Essential dimensions of sealing systems.

Figure 2 displays the essential dimensions (in mm) of coaxial sealing systems of cylinder pistons.

## 3. Study of Friction in Coaxial Sealing Systems

The assembly consisting of the seal, the inner surface of the cylinder barrel and the working fluid (hydraulic oil) represents the friction couple that is analyzed further on. A smooth and jerk-free motion of the piston at small velocities can be achieved by ensuring an optimum roughness of the contacting surfaces, entailing the deployment of materials with the smallest friction coefficients.

In [20], the authors published a study on the influence of the friction couple components on the motion of the piston. In accordance with the laws of hydro-dynamic lubrication, with the onset of piston motion, the initially contacting surfaces of the cylinder barrel and the seal are separated by a fluid film of a thickness that depends on the velocity, thus ensuring the targeted jerk-free motion. An experimental study has revealed that such beneficial hydro-dynamic friction can be attained even if the average fluid thickness of the fluid film is smaller than the *Ra* roughness sum of the two surfaces (*Ra* is the arithmetic average of the absolute values of the profile height deviations from the mean line, recorded within the evaluation length).

A jerk-free motion can be obtained also by selecting low friction materials for the seals. In the literature, however, different friction coefficient values are mentioned for the same pair of contacting materials. This can be explained by the fact that when mentioning that different values are concrete conditions for determining, these values are not detailed, such as apparatus and methodology, or the values and orientation of contacting surface roughness. For these reasons, it is advisable to determine friction coefficients by measurements conducted under specified conditions, rather than using technical calculation values taken from the literature, determined under unknown conditions.

In view of the above assertions, the requirement becomes evident of experimentally determining the friction coefficients between the seal and its contact surface with maximum possible accuracy and a good reproducibility of results. Continuing, this paper presents the methodology for determining the static and kinematic friction coefficients for the pairs of materials enumerated above.

### 3.1. Determination of the Static Friction Coefficient

The resisting force that hinders the displacement of an object on a smooth and hard surface is known as the friction force. This depends on the deformability and the roughness of the materials of the two contacting surfaces. The static friction coefficient (*μ_s_*) is defined as the ratio of the maximum static friction force between the contacting surfaces prior to the onset of the motion (*F_fr s_*) and the normal force (*N*).

(1) $$\mu_{s}\;=\;\frac{F_{fr\;s}}{N}$$

**Figure 3:**
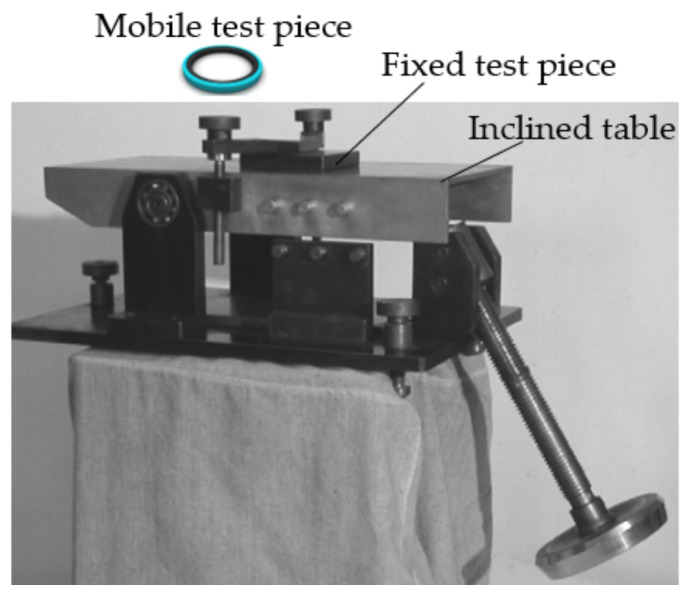
High precision tribometer.

The values of the static friction coefficients were determined by means of a tribometer operating on the principle of linear measurement (Figure 3) [21].

Underlying the design of the tribometer is the concept of the inclined plane that links the tilting angle *α* and the static friction coefficient *µ_s_*. The tested assembly, initially stationary, is tilted until the mobile test piece slides over the fixed one. Equation (2) shows the correlation of the angle *α* at that sliding occurs with the static friction coefficient:

(2) $$\mu_{s}\;=\;\tan\alpha$$

Experiments were conducted in order to determine the static friction coefficients in the following pairs of materials: OLC45/Virgin PTFE, OLC45/PTFE 25% Glass, and OLC45/H-PU 55D, under certain machining conditions and at different loads. The test pieces have the following characteristics:

- The fixed test pieces are made from OLC45 with a roughness *Ra* of the contacting surfaces ranging from 0.1 to 0.8 µm;
- The mobile test pieces are the seals themselves with the following roughness: for Virgin PTFE: *Ra* = 3.2 µm; for PTFE 25% Glass: *Ra* = 2.57 µm; for H-PU 55D: *Ra* = 3.2 µm.

**Figure 4:**
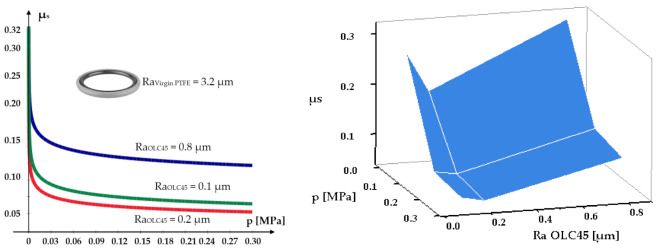
Dependency of the static friction coefficient on contact pressure and surface roughness for the material pair OLC45/Virgin PTFE.

**Figure 5:**
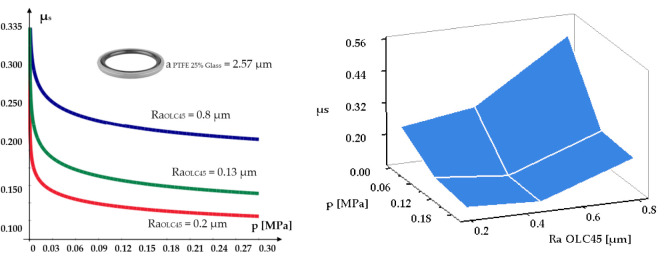
Dependency of the static friction coefficient on contact pressure and surface roughness for the material pair OLC45/PTFE 25% Glass.

**Figure 6:**
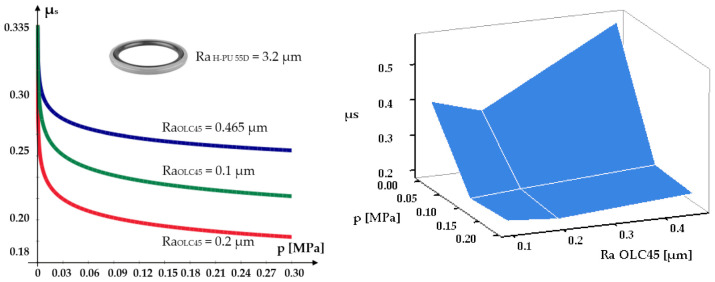
Dependency of the static friction coefficient on contact pressure and surface roughness for the material pair OLC45/H-PU 55D.

The static friction coefficients were determined by loading each mobile test piece with weights, thus varying the pressure on the fixed test piece between 0 and 0.3 MPa. For each pair of tested materials, the curves and surfaces were plotted, indicative of the dependency of the static friction coefficient on the roughness of the contacting components and the pressure. Figure 4, Figure 5 and Figure 6 present the dependencies of the static friction coefficients on load pressure and surface roughness of the two contacting materials.

**Figure 7:**
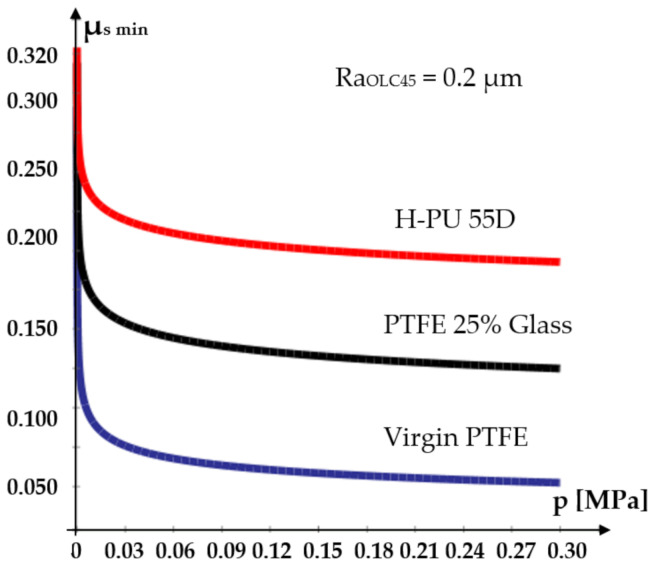
Dependency of the minimum static friction coefficient on contact pressure and seal material.

An analysis of the figures yields a number of important conclusions regarding the variation of the static friction coefficients, depending on the used materials and the contacting surfaces roughness. Thus:

- The smallest value of the static friction coefficient is generated by Virgin PTFE (*μ_smin_* = 0.048);
- For Virgin PTFE and PTFE 25% Glass, the values of the static friction coefficients are smaller than for H-PU 55D. This is due to the existence of large and negatively charged fluorine atoms that neutralize the positive charge of the carbon atoms. This facilitates easy sliding of the molecule chains, one in relation to another. Additionally, responsible for good friction behavior is the layered structure of PTFE, resembling that of solid lubricants [22].
- As the load of the tribosystem increases, regardless of the analyzed material, the value of the static friction coefficient decreases to a minimum, where it remains relatively constant. This behavior is explained by the fact that at zero load, the molecule chains at the surface of the test piece do not slide one in relation to another, the micro-asperities having to climb over the encountered obstacles (rough metallic surface). A phenomenon of reciprocal hooking of the two components occurs, causing a high friction coefficient. As the load grows, the links between the molecule chains grow weaker, allowing relatively easy sliding, directly causing the static friction coefficient to diminish.
- The roughness of the surface over which the seal glides also influences the magnitude of the coefficient *μ_s_*. The obtained results show that *μ_s_* reaches a minimum for a certain value of the roughness of this surface. For all seal materials the smallest values of the static friction coefficient were obtained for a fixed test piece roughness of *Ra* = 0.2 µm. For this situation, Figure 7 presents, comparatively, the evolution of the static friction coefficients.

For roughness values exceeding 0.2 µm, the friction coefficients increase. This behavior can be explained by the extra effort required from the elements of the tribosystem to overcome the obstacles created by the larger asperities of the contacting surfaces.

### 3.2. Determination of the Kinetic Friction Coefficient

A kinematic friction force is generated at the contact between two bodies that slide one over another. This force is determined by the chemical bonds between the materials of the two contacting surfaces and by their asperities. The kinetic friction coefficient *μ_k_* is defined as the ratio between the maximum kinematic friction force (*F_fr k_*) and the normal force (*N*) to the surface over which the movable body slides.

(3) $$\mu_{k}\;=\;\frac{F_{fr\;k}}{N}$$

**Figure 8:**
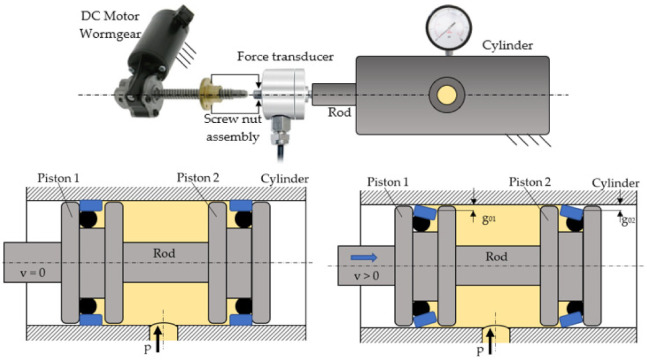
Construction of the test stand for the determination of the kinetic friction coefficient.

Experiments were conducted in order to obtain the values of the kinematic friction coefficients indirectly, by previously determining the friction forces generated in coaxial sealing systems. The conceived experimental test stand consisted of an electromechanical system for the generation of the translation motion and a hydraulic cylinder of special construction, the mobile assembly of which includes a rod and two pistons mounted and fixed together. The two pistons ensure the pressure on the working fluid in the chamber (Figure 8).

As the coaxial sealing systems are tested in pairs, the hydraulic cylinder is equipped with two identical pistons enclosing the pressurized fluid between them [20]. This is a balanced construction from the viewpoint of the forces, as the pressurized fluid cannot displace the assembly of the two pistons. The motion of the mobile assembly consisting of the two pistons is generated by a direct current motor equipped with a reduction worm gear and connected to a screw mechanism. The displacement of this mechanism is opposed only by the kinematic friction force generated by the two coaxial sealing systems, measured by means of a resistive transducer. With the onset of motion two wedge-shaped fluid films are formed, one for each piston, in the contact area of the respective seals with the cylinder barrel. The average thicknesses of the fluid films are not identical (*g_01_* and *g_02_,* respectively) and the method for computing these is described in [4]. The fluid film thicknesses, being unequal, yields the conclusion that the kinematic friction forces generated by the two seals are not equal either.

The utilized force transducer measures the sum of the two kinematic friction forces generated by the tested seals (*F_fr k tot_mes_*).

(4) $$F_{fr\;k\;tot\_mes}\;=\;F_{fr\;k1}+F_{fr\;k2}$$

As the individual kinematic friction forces are not equal, the value of each is determined by a different equation [20]:

(5) $$F_{fr\;k1}\;=\;\pi \times D\times b\times \left[p\times \mu_{k}\times \beta +2\times \left(1-\beta \right)\times ln\frac{1}{\beta }\times \frac{\eta \times v}{g_{01}}\right]$$

(6) $$F_{fr\;k2}\;=\;\pi \times D\times b\times \left[p\times \mu_{k}\times \beta +2\times \left(1-\beta \right)\times ln\frac{1}{\beta }\times \frac{\eta \times v}{g_{02}}\right]$$

with the following notations: *D* = exterior diameter of the seal (60 mm); *b* = seal width (4 mm); *p* = pressure to be sealed off; *μ_k_* = kinetic friction coefficient; *β* = real non-dimensional contact surface area (computed as the ratio of the real and the nominal surface area between the seal and the sealed-off surface); *η* = dynamic viscosity of the working fluid (Anti-wear Hydraulic Oil ISO VG 32); *v* = velocity of displacement; *g*_01,2_ = average magnitudes of the gaps formed between the seals and their adjacent surfaces.

The non-dimensional real area *β* depends on the pressure exerted by the seal onto the cylinder barrel. Table 2 shows the values of *β* for the three studied materials [4,20]:

The expression of the kinetic friction coefficient can be extracted from Equations (4)–(6):

(7) $$\mu_{k}\;=\;\frac{1}{\pi \times D\times b\times p\times \beta }\times \left[\frac{F_{fr\;k\;tot\_mes}}{2}-\left(1-\beta \right)\times ln\frac{1}{\beta }\times \pi \times D\times b\times \eta \times v\times \left(\frac{1}{g_{01}}+\frac{1}{g_{02}}\right)\right]$$

**Figure 9:**
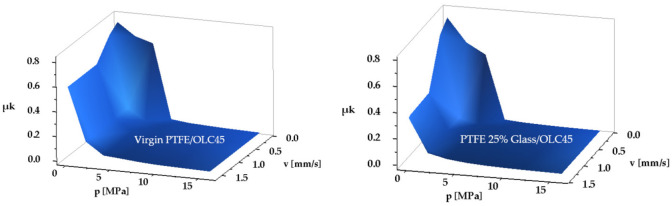
Dependency of the kinematic friction coefficient on contact pressure and velocity.

In order to measure the friction forces, the fluid volume between the two pistons was subjected to pressures up to 16 MPa, while the velocities ranged from 0.125 to 1.6 mm/s. Table 3 presents the results obtained for the material pairs, Virgin PTFE/OLC45 and PTFE 25% Glass/OLC45, and Figure 9 shows the surfaces that describe the variation of the kinematic friction coefficient versus contact pressure and velocity:

The following conclusions emerge:

- As fluid pressure increases; the kinematic friction coefficient values fall at a larger rate for small pressures (0–4 MPa) and at a lower rate at higher pressures. Notably the values of the kinematic friction coefficients for PTFE 25% Glass are smaller than for Virgin PTFE. This can be explained by the different yield points of the two materials, that lead to unequal real non-dimensional contact surface areas for the same load. The higher *β* is (the case of PTFE 25% Glass), the smaller the kinematic friction coefficient will be.
- As the velocities decrease, the kinematic friction coefficients have greater values, and can cause jerky motions. This behavior is evident for small pressures (0–4 MPa), while at high pressures (>4 MPa) a relative independence of the friction coefficient on the velocity can be observed.

**Figure 10:**
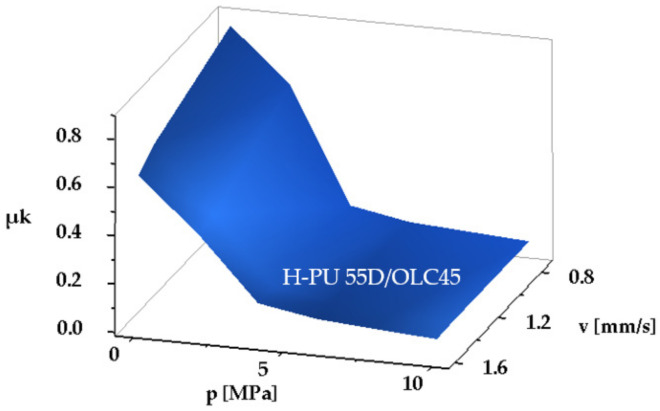
Dependency of the minimum kinematic friction coefficient on contact pressure and velocity for the material pair H-PU 55D/OLC45.

The seal material with the greatest yield point is H-PU 55D, hence the real non-dimensional contact surface area *β* is smaller. Determination of the kinematic friction coefficients was possible only at large velocities, over 0.745 mm/s, as up to this threshold the motion is affected by stick-slip and/or eigen-vibrations. Table 4 shows the results obtained for the pair of materials H-PU 55D/OLC45, and Figure 10 illustrates the dependency of the minimum kinematic friction coefficient on contact pressure and velocity:

To be noted is that this pair of materials generates the largest kinematic friction coefficient, thus entailing high energy inefficiency.

A universal oscilloscope was used to visualize the pulsatory character of the friction force. The resulting oscillograms allowed conclusions concerning the moment of onset of the friction force pulsations.

The behavior of Virgin PTFE and PTFE 25% Glass is similar. Stick-slip occurred at small pressures (<4 MPa) and at velocities under 0.2 mm/s, followed by eigen-vibrations up to 0.3 mm/s. In the case of H-PU 55D, stick-slip is apparent over the entire range of small pressures up to a velocity of 1.4 mm/s. The eigen-vibrations manifest up to 1.5 mm/s. Beyond this, the velocity displacement is uniform and pulsation-free. Regardless of the velocity, for all three studied materials, a uniform motion was obtained as soon as the fluid pressure exceeded 4 MPA.

**Figure 11:**
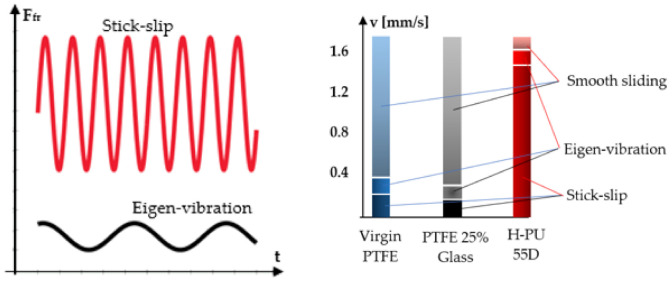
Critical velocity values for the onset of stick-slip and eigen-vibrations by seal material.

Figure 11 presents by seal material the critical velocities where stick-slip and eigen-vibrations occur (*p* < 4 MPa).

The figure above highlights materials Virgin PTFE and PTFE 25% Glass as optimum for seals used in small velocity applications. A smooth motion of cylinder pistons equipped with seals made from H-PU 55D can be achieved for velocities above 1.6 mm/s.

## 4. Conclusions

Energy efficiency of hydraulic cylinders requires inter alia the diminishing of friction forces, achievable by an adequate selection of seal materials. The most performant materials are polymers, such as polytetrafluoroethylene (PTFE) or polyurethan. The paper presents an analysis of the friction-related behavior of three polymer materials used for the seals of hydraulic cylinder pistons. The tested materials were the thermoplastic polymers Virgin PTFE and PTFE 25% Glass, and H-PU 55D from the category of Polyurethan.

The results presented and discussed in this paper highlight the friction-related behavior of polymer compounds used in the construction of coaxial sealing systems. Experiments were conducted to determine the values of the static and kinematic friction coefficients, ensued by recommendations as to the most adequate materials for hydraulic cylinder seals.

The experimental research yielded a number of significant conclusions:

- The optimum roughness of the hydraulic cylinder barrel is *Ra* = 0.2 μm;
- For Virgin PTFE and PTFE 25% Glass the values of the static friction coefficients are smaller than for H-PU 55D (the smallest value of the static friction coefficient is generated by Virgin PTFE);
- Regardless of the analyzed material, the value of the static friction coefficient decreases to a minimum, as the load of the tribosystem increases;
- The value of the kinematic friction coefficient decreases as the working fluid pressure grows (the smallest value of the kinematic friction coefficient is generated by PTFE 25% Glass);
- Kinematic friction coefficient values increase as the velocity falls;
- The moment of jerky motion onset (stick-slip or eigen-vibrations) depends on the velocity, the seal material and the pressure of the working fluid.

In order to avoid stick-slip or eigen-vibrations at small velocities the results of the discussed research recommend the use of PTFE polymers for seals.

## Figures and Tables

**Table 1:** Characteristics of seal materials.

Material	Composition	Shore D Hardness	Utilization
Virgin PTFE	100% PTFE	55 ± 3	Resistant to almost all chemicals
PTFE 25% Glass	25% clean milled glass fibers and 75% virgin PTFE	58 ± 3	Resistant to almost all chemicals
H-PU 55D	PUR	55 ± 3	Resistant to oil, petrol, hot water, hot air, ozone

**Table 2:** Values of the non-dimensional contact surface area *β*.

Pressure *p* [MPa]							
0	2	4	6	8	10	12	16
Non-dimensional contact surface area *β* for Virgin PTFE [%]							
2.5	9.2	15.9	22.5	29.2	35.9	42.5	55.9
Non-dimensional contact surface area *β* for PTFE 25% Glass [%]							
4.3	15.8	27.2	38.6	50	61.5	72.9	95.8
Non-dimensional contact surface area *β* for H-PU 55D [%]							
2.3	8.2	14.2	20.2	26.1	32.1	38.1	50

**Table 3:** Dependency of *μ_kmin_* on contact pressure and velocity.

**Virgin PTFE/OLC45**					
Pressure [MPa]	Velocity [mm/s]				
	1.6	1.455	0.745	0.355	0.125
0	0.591	0.591	0.591	0.7502	0.8023
2	0.1614	0.1652	0.1652	0.1689	0.7012
4	0.0623	0.0623	0.0752	0.0765	0.6575
6	0.0508	0.0542	0.0551	0.0556	0.0563
8	0.0376	0.0389	0.0390	0.0392	0.0397
10	0.0291	0.0297	0.0297	0.0308	0.0323
12	0.0247	0.0249	0.0249	0.0252	0.0258
16	0.0169	0.0169	0.0172	0.0172	0.0173
**PTFE 25% Glass/OLC45**					
Pressure [MPa]	Velocity [mm/s]				
	1.435	1.389	0.755	0.377	0.136
0	0.343	0.353	0.363	0.7111	0.7805
2	0.0872	0.0891	0.0909	0.0909	0.6023
4	0.0528	0.0543	0.0544	0.0551	0.5210
6	0.0362	0.0366	0.0370	0.0370	0.0382
8	0.0266	0.0266	0.0268	0.0268	0.0271
10	0.0210	0.0210	0.0212	0.0214	0.0217
12	0.0168	0.0169	0.0169	0.0171	0.0171
16	0.0112	0.0113	0.0114	0.0115	0.0115

**Table 4:** Dependency of *μ_kmin_* on contact pressure and velocity (H-PU 55D/OLC45).

H-PU 55D/OLC45			
Pressure [MPa]	Velocity [mm/s]		
	1.6	1.455	0.745
0	0.6330	0.6950	0.8540
2	0.4153	0.4261	0.6320
4	0.1523	0.1523	0.1563
6	0.1103	0.1112	0.1118
8	0.0921	0.0934	0.0956
10	0.0759	0.0797	0.0803

## Data Availability

The data presented in this study are available in this article and in [4,20].
